# Balanced-BiEGCN: A Bidirectional EvolveGCN with a Class-Balanced Learning Network for Dynamic Anomaly Detection in Bitcoin

**DOI:** 10.3390/e27101045

**Published:** 2025-10-08

**Authors:** Bo Xiao, Wei Yin

**Affiliations:** School of Economics and Management, Southeast University, Nanjing 211189, China; kellie93@126.com

**Keywords:** dynamic graph, imbalanced data, anomaly detection, Bitcoin, feature fusion

## Abstract

Bitcoin transaction anomaly detection is essential for maintaining financial market stability. A significant challenge is capturing the dynamically evolving transaction patterns within transaction networks. Dynamic graph models are effective for characterizing the temporal evolution of transaction systems. However, current methods struggle to mine long-range temporal dependencies and address the class imbalance caused by the scarcity of abnormal samples. To address these issues, we propose a novel approach, the Bidirectional EvolveGCN with Class-Balanced Learning Network (Balanced-BiEGCN), for Bitcoin transaction anomaly detection. This model integrates two key components: (1) a bidirectional temporal feature fusion mechanism (Bi-EvolveGCN) that enhances the capture of long-range temporal dependencies and (2) a Sample Class Transformation (CSCT) classifier that generates difficult-to-distinguish abnormal samples to balance the positive and negative class distribution. The generation of these samples is guided by two loss functions: the adjacency distance adaptive loss function and the symmetric space adjustment loss function, which optimize the spatial distribution and confusion of abnormal samples. Experimental results on the Elliptic dataset demonstrate that Balanced-BiEGCN outperforms existing baseline methods in anomaly detection.

## 1. Introduction

Bitcoin is widely considered the first decentralized digital currency [1], relying on a consensus mechanism and distributed architecture to ensure transaction transparency and stability. While its anonymity [2] protects user privacy, it also enables illicit activities such as money laundering and extortion [3,4,5,6]. These activities not only pose financial risks but also complicate regulatory oversight, making anomaly detection essential for identifying fraud by uncovering hidden illegal transaction patterns.

Recent advances in machine learning [7] and neural networks [8] have significantly improved detection techniques. Bitcoin transaction data, represented as a dynamic graph, evolves over time through correlated subgraphs, motivating research into dynamic graph anomaly detection (DGAD) [9]. In static graph scenarios, graph neural networks (GNNs) [10] have been effective for node classification and edge prediction. In dynamic settings, the integration of graph convolutional networks (GCNs) with temporal feature modeling [11,12,13] has enhanced feature extraction for Bitcoin transaction graphs. Notably, EvolveGCN [14] adapts GCN parameters dynamically through recurrent neural networks (RNNs) [15], capturing evolving graph structures and node attributes. However, EvolveGCN’s unidirectional temporal modeling limits its ability to capture long-range temporal dependencies in transaction data.

Moreover, Bitcoin transaction networks suffer from severe class imbalance, as abnormal transactions are rare compared to normal transactions. This imbalance leads models to favor the majority class, reducing detection accuracy for anomalies. Current solutions, such as GraphSMOTE [16], address this issue by generating synthetic samples based on node connectivity; however, they struggle with detecting subtle anomalies whose spatial distributions overlap with those of normal nodes.

To overcome these challenges, we propose the Bidirectional EvolveGCN with Class-Balanced Learning Network (Balanced-BiEGCN) for Bitcoin transaction anomaly detection. The model consists of two key innovations: (1) Bi-EvolveGCN, a bidirectional temporal feature fusion mechanism that enhances the capture of long-range dependencies in dynamic graphs, and (2) a Sample Class Transformation Classifier (CSCT), which balances class distributions by generating difficult-to-distinguish abnormal samples. The generation of these samples is guided by two novel loss functions: (a) the Adjacency Distance Adaptive Loss function, which optimizes the adjacency relationships of generated anomalies to mimic real abnormal nodes, and (b) the Symmetric Space Adjustment Loss function, which adjusts the spatial distribution of hard-to-detect anomalies to improve detection performance.

In summary, the contributions of this paper are:

1. We design Balanced-BiEGCN, a novel framework that integrates Bi-EvolveGCN for bidirectional temporal feature extraction and CSCT to address class imbalance through synthetic anomaly sample generation.
2. We propose the Adjacency Distance Adaptive Loss function to align the topological properties of synthetic anomalies with those of real anomalous nodes, thereby enhancing anomaly detection.
3. We develop the Symmetric Space Adjustment Loss function to improve the detection of subtle anomalies by refining their spatial distribution.

## 2. Related Work

Bitcoin, due to its decentralized and encrypted nature, has become an innovative medium for financial transactions. However, its anonymity and immutability also enable fraud and illegal transactions, making Bitcoin transaction anomaly detection a crucial area of research in fintech. This task involves analyzing blockchain records to extract graph structural features, node attributes, and temporal dynamics, to detect anomalous transactions or malicious entities [17].

Current detection methods can be broadly classified into two categories: traditional methods based on feature engineering [18] and methods leveraging deep learning [19,20]. Traditional approaches focus on manually designing features such as transaction frequency, amount, and node connectivity, using classical machine learning algorithms like linear regression and decision trees for classification. In contrast, deep learning-based methods, particularly Graph Neural Networks (GNNs) and self-supervised learning frameworks, automatically learn latent feature representations from transaction networks, significantly improving detection accuracy and robustness [21,22,23].

Despite these advances, significant challenges remain, including the scarcity of labeled anomalies and the increasing concealment of malicious activities. Dynamic graph anomaly detection (DGAD), which focuses on identifying anomalies in evolving graph structures, introduces further complexities, including dynamic topology modeling, temporal dependency capture, and robustness to noise. In the Bitcoin transaction context, the dynamic nature of transaction relationships requires temporal graph models for accurate representation. Notable approaches include DyGCN [24], which updates GCN parameters dynamically using LSTM [25] and mutual information maximization to capture global structures, enabling the identification of abnormal address-node connections in the transaction network. PI-GNN [26] addresses catastrophic forgetting by freezing stable subgraph parameters and expanding new ones to capture emerging abnormal behaviors. STRIPE [27] decouples transaction features into steady-state topologies (e.g., transaction communities) and dynamic attributes (e.g., single transaction amounts), using memory networks and mutual attention mechanisms to detect anomalies.

Further advancements in spatiotemporal joint modeling and efficiency optimization include DGL-LS [28], which uses multi-scale convolutional kernels and attention gates to capture both short-term and long-term temporal dependencies, thus identifying large transfers and abnormal trends. Dy-SIGN [29] employs spiking neural networks and implicit differentiation, representing transactions as binary spikes and reducing memory consumption to half that of traditional methods.

Existing research in Bitcoin transaction anomaly detection has made notable progress, especially with deep learning methods like Graph Neural Networks (GNNs) and self-supervised learning, which have enhanced detection accuracy. However, challenges remain in modeling the dynamic and temporal aspects of transaction networks, as well as handling class imbalance and the scarcity of labeled anomaly samples. While models like DyGCN and PI-GNN have made strides in addressing dynamic challenges, capturing long-term temporal dependencies and evolving anomalous behaviors remains an open area for improvement. This paper aims to bridge these gaps by proposing a novel model that integrates bidirectional temporal feature integration with class imbalance techniques, enhancing the detection of subtle and emerging anomalies. The proposed approach enhances long-term temporal dependency modeling and offers a robust framework for handling dynamic and complex transaction behaviors, thereby providing a more comprehensive solution to the outstanding challenges in this field.

## 3. Method

**Figure 1:**
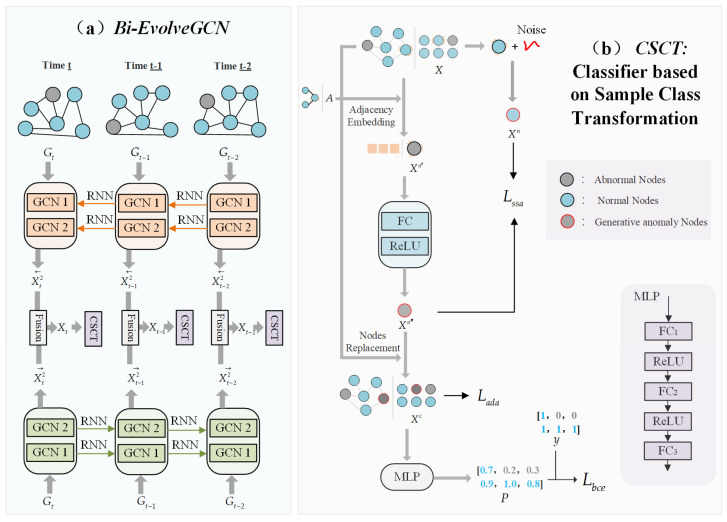
Balanced-BiEGCN: (**a**) First, Bi-EvolveGCN extracts the temporal change characteristics of dynamic transaction graphs from dynamic graph G, and captures the bilateral temporal dependencies between nodes through bidirectional temporal subgraph embedding; (**b**) CSCT converts some normal nodes into new abnormal node classes to alleviate the sample category imbalance problem.

This paper proposes a Bidirectional EvolveGCN with a Class-Balanced Learning Network (Balanced-BiEGCN) to address the challenge of anomaly detection in dynamic Bitcoin transaction graphs. The entire method consists of two stages: node feature extraction and classification. First, in the feature extraction stage, as shown in Figure 1a, the Bi-EvolveGCN module learns node representations by aggregating information from both forward and backward temporal subgraphs. Technical details are provided in Section 3.1. Second, in the classification stage, as shown in Figure 1b, the CSCT balances the class distribution by synthesizing hard-to-distinguish anomalous samples from normal ones. Technical details are given in Section 3.2. Furthermore, Section 3.3 details the loss functions of Balanced-BiEGCN. The adjacency distance adaptive loss and the symmetric space adjustment loss are core constraint mechanisms, providing critical guidance during the synthesis of a new anomalous node class.

To model the dynamic nature of Bitcoin transaction graphs, the Balanced-BiEGCN decomposes the input data into a sequence of *T* temporal subgraphs, each representing a specific time window. Formally, a snapshot at time point t is denoted as $G_{t}=(V_{t},A_{t})$, where $V_{t}\in R^{N}$ represents the node set (N is the number of nodes); $A_{t}\in R^{N\times N}$ is the adjacency matrix, where $A_{t}\left[i,j\right]=$1 if an edge exists between nodes i and j, and 0 otherwise; $X_{t}\in R^{N\times d}$ represents the d_dimensional feature vectors of the nodes (e.g., containing transaction amount, timestamp, etc.).

### 3.1. Bi-EvolveGCN

**Figure 2:**
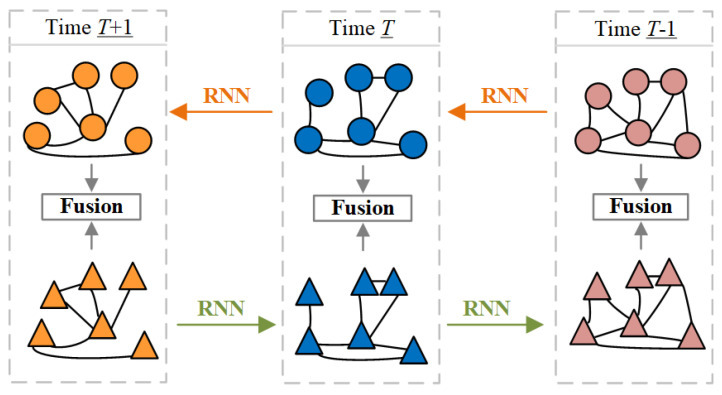
Shows the design principle of Bi-EvolveGCN, focusing on the modeling logic of dual temporal feature fusion.

In the feature extraction stage, Balanced-BiEGC designs a bidirectional temporal feature fusion extractor (Bi-EvolveGCN) based on EvolveGCN. As briefly shown in Figure 2, at three consecutive time points *T* − 1, *T*, and *T* + 1, Bi-EvolveGCN embeds forward-backward temporal subgraphs (to better distinguish the direction, the subgraph nodes are displayed as circles and triangles, respectively) into the feature graph. This design concept borrows the bidirectional temporal modeling mechanism of Bi-LSTM [30] and is particularly suitable for long-range dependency analysis tasks in dynamic graphs. Specifically, taking time point t as an example, the Bi-EvolveGCN in Figure 1a is composed of two bidirectional EvolveGCNs. The RNN temporal update network of EvolveGCN includes two versions, -O and -H, which differ in that they use LSTM and GRU [31] as their core units, respectively. LSTM excels at handling data scenarios with sparse node features but significant dynamic changes in graph structure, giving the -O version an advantage in Bitcoin transaction anomaly detection tasks. This critical speed advantage ensures that EvolveGCN-O can flag suspicious transactions before they are confirmed on the blockchain, whereas attention-based models, bogged down by computational overhead, often miss the narrow window for intervention. Therefore, this paper selects the -O version as the foundation for constructing Bi-EvolveGCN.

Bi-EvolveGCN employs a bidirectional recurrent update strategy to model dynamic graph temporal features, with each EvolveGCN comprising two consecutive GCNs. First, we introduce the forward and backward learnable weights $\overset{\leftarrow }{W_{t}^{l}}$ and $\overset{\to }{W_{t}^{l}}$ of the GCN at the *l*-th layer. The update rule for $\overset{\leftarrow }{W_{t}^{l}}$ is as follows:

(1) $$\overset{\leftarrow }{W_{t}^{l}}=\mathrm{LSTM}\left(\overset{\leftarrow }{W_{t-1}^{l}}\right)$$

The above is a forward weight update, which applies LSTM (a special variant of RNN) to capture forward temporal dependencies, inputs the weight at time t−1 into LSTM, and learns the long-term temporal relationships of node features. Similarly, the reverse weight $\overset{\to }{W_{t}^{l}}$ is updated from $\overset{\to }{W_{t+1}^{l}}$. Next, based on $\overset{\leftarrow }{W_{t}^{l}}\in R^{d_{l-1}\times d_{l}}$, perform the forward update operation of node feature $V_{t}$. Taking the forward node feature $\overset{\leftarrow }{X_{t}^{l}}$ output by the *l*-th layer of GCN as an example, the update formula is:

(2) $$\overset{\leftarrow }{X_{t}^{l}}=ReLU\left(A_{t}\overset{\leftarrow }{X_{t}^{l-1}}\overset{\leftarrow }{W_{t}^{l}}+\overset{\leftarrow }{b_{t}^{l}}\right)$$

Among them, ReLU(·) stands for the ReLU activation function [32], and nonlinear factors can be introduced to enhance the expressive power of the model. $A_{t}\in R^{N\times N}$ is the adjacency matrix of the subgraph at time t. $\overset{\leftarrow }{X_{t}^{l-1}}\in R^{N\times d_{l-1}}$ (if l=1, then $\overset{\leftarrow }{X_{t}^{l-1}}=V_{t}$) is the positive node feature of layer l−1, and $\overset{\leftarrow }{b_{t}^{l}}\in R^{1\times d_{l}}$ is the intercept. The final output of the forward node feature is $\overset{\leftarrow }{X_{t}^{2}}$. Similarly, $\overset{\to }{X_{t}^{2}}$ is the final output of the reverse node feature. Finally, $\overset{\leftarrow }{X_{t}^{2}}$ and $\overset{\to }{X_{t}^{2}}$ are merged to obtain the final node feature $X_{t}$

(3) $$X_{t}=\mathrm{Con}\left(\left(\overset{\leftarrow }{X_{t}^{2}},\overset{\to }{X_{t}^{2}}\right),1\right)$$

The above formula Con((a,b),c) concatenates features a and b along dimension c. The above process fuses bidirectional temporal information into node features, providing support for long-time distance relationship mining in subsequent tasks.

### 3.2. Classifier Based on Sample Class Transformation

The CSCT method uses existing abnormal nodes as references and generates new abnormal nodes based on partial normal nodes from the perspectives of adjacency and spatial distribution. As shown in Figure 1a, teach temporal subgraph deploys a CSCT to perform node classification tasks. Figure 1b take an arbitrary temporal subgraph as an example, the node features input to CSCT are denoted as $X\in R^{N\times d}$. Here, N is the total number of nodes in the graph, and *A* is the dimension of a single node feature.

First, randomly select normal nodes $X^{a}$ (numbered as $I^{a}$) with a ratio of ε as the basis for conversion to new abnormal nodes. Next, simultaneously perform neighbor node embedding on $X^{a}$ to obtain ${X^{a}}^{\prime }$ and add noise to obtain $X^{n}$. The formulas are as follows:

(4) $${X^{a}}^{\prime }=\left(A\left[I^{a},:\right]\right)\times X^{a}$$

(5) $$X^{n}=X^{a}\left[I^{a},:\right]+\left(\mathrm{Ran}\left(X^{a}\left[I^{a},:\right].\mathrm{size}\right)\times \sigma^{2}+\mu \right)$$

Among them, $A\in R^{N\times N}$ is the node adjacency matrix; Ran(a.size) is used to generate a normal distribution random number matrix consistent with the feature a dimension, and the mean μ and variance $\sigma^{2}$ jointly constrain the specific normal distribution that the noise obeys. This noise addition constructs a symmetric feature space that is close to normal node features and difficult to distinguish, which can provide a spatial distribution guide for the generation process of novel abnormal nodes. Subsequently, ${X^{a}}^{\prime }$ undergoes feature transformation through a fully connected (FC) layer and a rectified linear unit (ReLU) layer, and the transformed results are replaced back into the corresponding node features of the original node set X with the numbered $I^{a}$. The formula is as follows:

(6) $$X^{c}=\mathrm{Rep}\left(\left(X,\mathrm{ReLU}\left(\mathrm{FC}\left({X^{a}}^{\prime }\right)\right)\right),I^{a}\right)$$

In this process, Rep((a,b),c) represents replacing the node features in set a whose IDs belong to c with the corresponding node features in set b in order. Finally, a multi-layer perceptron (MLP) [33] consisting of three FC layers and two ReLU layers performs classification prediction on the replaced node feature set $X^{c}$, as shown in the following formula:

(7) $$P=\mathrm{MLP}\left(X^{c}\right)$$

MLP(·) is the multi-layer perceptron method, $P\in R^{N}$.

### 3.3. Loss Functions

To ensure that the novel generated abnormal nodes are close to the real abnormal nodes, CSCT designed the adjacency distance adaptive loss function and the symmetric space adjustment loss function to constrain the novel generated abnormal nodes to tend toward the actual ones. At the same time, the basic binary cross-entropy loss function [34] was introduced as the basic optimization objective for the classification task.

**Figure 3:**
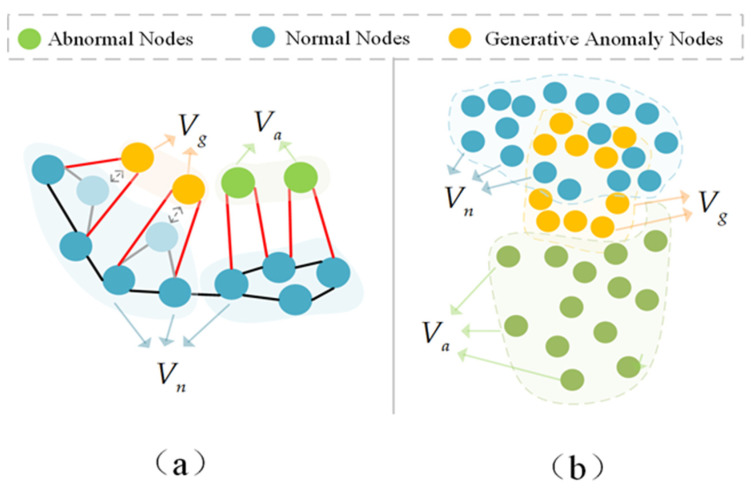
(**a**) shows the process of the adjacency distance adaptive loss function constraining the adjacent relationships of newly generated abnormal nodes; (**b**) demonstrates the guiding mechanism of the symmetric space adjustment loss function for generating the spatial distribution of new abnormal node classes.

Specifically, Figure 3 shows the design principle of the adjacency distance adaptive loss function and the symmetric space adjustment loss function, where $V_{n}$ is the normal node class, $V_{a}$ is the abnormal node class, and $V_{g}$ is the generated abnormal node class. The adjacency distance adaptive loss function is shown in Figure 3a, where $V_{n}$ is more closely related to the adjacent nodes, and $V_{a}$ is relatively loose. It aims to generate abnormal node class $V_{g}$ by adjusting the adjacent distance of some normal nodes so that they are closer to $V_{a}$ in terms of adjacent relationships. The symmetric space adjustment loss function is shown in Figure 3b. The spatial distribution of difficult-to-distinguish abnormal node classes is closer to $V_{n}$ than to the whole $V_{a}$, aiming to guide $V_{g}$ to tend toward $V_{n}$ in terms of spatial distribution.

#### 3.3.1. Adjacency Distance Adaptive Loss Function

In order to map the adjacency relationship of existing abnormal nodes to the generation of novel abnormal nodes, CSCT designed an adjacency distance adaptive loss function to guide the aggregation degree of novel generated abnormal nodes and their neighboring nodes to be close to that of real abnormal nodes.

In the specific implementation, first, the feature matrix $X^{c}$ of the initial node set is normalized. When there is an edge connection between nodes i and j, the similarity matrix $S_{i,j}$ between the two is constructed by element-wise inner product computation, and the calculation formula is as follows:

(8) $$S_{i,j}=\sum_{k=1}^{N}\frac{X_{ik}^{c}}{\sqrt{\sum_{l=1}^{d}{X_{il}^{c}}^{2}}}\times \frac{X_{jk}^{c}}{\sqrt{\sum_{l=1}^{d}{X_{jl}^{c}}^{2}}}$$

Next, based on $S_{i,j}$, calculate the aggregation degree of each node in the adjacency relationship using the following formula:

(9) $$F_{i}=\frac{\sum_{j=1}^{N}S_{i,j}}{\sum_{j=1}^{N}A_{i,j}}(i=1,2,\cdots ,N)$$

In the above formula, $\sum_{j=1}^{N}A_{i,j}$ calculates the number of adjacent nodes of node i in the graph structure based on the graph adjacency matrix A. The higher the value of $F_{i}$, the closer the association between node $F_{i}$ and other nodes.

Then, the adjacency distance adaptation loss $L_{ada}$ is obtained by the following formula:

(10) $$L_{ada}=\max(0,\left|\frac{1}{N^{a}}\sum_{i\in I^{a}}F_{i}-\frac{1}{N^{b}}\sum_{i\in I^{b}}F_{i}\right|)$$

In the above formula, when x=a, the affinity mean of the real abnormal node class is calculated, $N^{a}$ is the number of real abnormal nodes, and $I^{a}$ is the index of the real node in the overall node set. Similarly, when x=b, the affinity mean of the abnormal nodes is calculated, corresponding to $N^{b}$ and $I^{b}$. $L_{ada}$ aims to reduce the difference in the degree of adjacency between the old and novel abnormal node classes.

#### 3.3.2. Symmetric Space Adjustment Loss Function

In dynamic graph anomaly detection tasks, pseudo-anomalous nodes generated by traditional adjacency aggregation often have feature space symmetry deficiencies—their distribution is difficult to approximate the high similarity boundary region between real abnormal nodes and normal nodes (i.e., the difficult-to-distinguish category boundary region). To solve this problem, this paper proposes the symmetric space adjustment loss function $L_{ssa}$, which models subtle differences in the feature space to strengthen the model’s ability to learn complex decision boundaries. The specific formula is as follows:

(11) $$L_{ssa}=\frac{1}{N^{a}}\sum_{i=1}^{N^{a}}\sqrt{\sum_{j=1}^{d}({X_{ij}^{a}}^{\prime \prime }-X_{ij}^{o}{)}^{2}}$$

The specific operation of the above formula is as follows: first, use the square sum of feature differences to amplify subtle deviations, thereby capturing hard-to-distinguish nodes; second, integrate global differences by taking the square root; finally, take the average value of the difference values of the new abnormal nodes, thereby guiding the model to focus on highly similar abnormal nodes.

#### 3.3.3. Basic Loss Function

In addition to the $L_{ada}$ and $L_{ssa}$ loss functions, Balanced-BiEGCN introduces a binary cross-entropy loss function as the basic classification loss, as shown in the following formula:

(12) $$L_{bce}=\sum_{i=1}^{N}w_{1}y_{i}\log(P_{i})+w_{0}(1-y_{i})\log(1-P_{i})$$

where $P_{i}$ is the probability that the node output from the classifier belongs to the normal category; $y_{i}$ is the node label (normal node $y_{i}=1$, abnormal node $y_{i}=0$); and $w_{1}$ and $w_{0}$ are the category weights, which are used to balance the loss contribution of positive and negative samples. Finally, to synthesize the effects of $L_{bce}$, $L_{ada}$, and $L_{ssa}$ on model training, the hyperparameter $l\in \left[0,1\right]$ weighted combination is used to balance three loss functions, and the total loss formula is:

(13) $$L=L_{bce}+l\cdot L_{ada}+(1-l)L_{ssa}$$

## 4. Experiments

### 4.1. Data and Set

Elliptic [35] is the largest publicly available labeled dataset in the domain of Bitcoin transaction analysis, containing 49 consecutive temporal subgraphs. The dataset is divided into training (1–30), validation (31–35), and test sets (36–49) based on the node data corresponding to the timestamps. During the training and testing phases, only the labeled nodes and their associated edges are utilized. The dataset contains a total of 203,769 nodes, of which 4545 are labeled as illicit (invalid) and 42,019 as licit (valid), along with 234,355 edges representing transaction relationships.

Given that Bitcoin transaction anomaly detection is formulated as a binary classification problem, this study adopts Precision, Recall, and F1 as the primary performance evaluation metrics. The respective formulas are defined as follows:

(14) $$Precision=\frac{TP}{TP+FP}$$

(15) $$Recall=\frac{TP}{TP+FN}$$

(16) $$F1=2\times \frac{Precision\times Recall}{Precision+Recall}$$

In these equations, TP (true positives) denotes the number of correctly identified positive (illicit) samples; FP (false positives) refers to negative (licit) samples incorrectly classified as positive; and FN (false negatives) indicates positive samples that are misclassified as negative.

### 4.2. Implementation

The experiments are implemented using the PyTorch 1.12.1 framework and executed on an NVIDIA RTX 3090 GPU. The model is optimized using the Adam optimizer with an initial learning rate set to 0.001. During the data sampling process, subgraphs are randomly selected from five consecutive temporal windows for each training iteration. The training process is conducted over 100 epochs.

To address the class imbalance inherent in the dataset, category weights are introduced in the loss function (as described in Equation (12)), with the majority and minority classes assigned weights of 0.35 and 0.65, respectively. This weighting scheme is designed to improve the model’s sensitivity to the minority class and enhance its performance on imbalanced classification tasks.

### 4.3. Comparison with State-of-the-Art Methods

Table 1 presents the experimental results of Balanced-BiEGCN and some other methods on the Elliptic dataset. To ensure the stability of the experiments, each method was tested 10 times independently, and the average value was taken as the final evaluation benchmark. The experimental results show that Balanced-BiEGCN outperforms other methods in all detection metrics, especially the F1 score, which reaches 0.765.

This section conducts comparative experiments from the perspectives of static graphs and dynamic graphs, selecting 10 mainstream methods including GCN and Skip-GCN. The core difference between the two lies in the way time information is embedded: static graphs integrate time as node features, while dynamic graphs divide continuous subgraphs based on time to capture temporal dependencies. In the static graph scenario, four methods—GCN, Skip-GCN, and others—belong to graph neural networks, with Skip-GCN and HHLN-GNN performing the best; Balanced-BiEGCN improves upon Skip-GCN and HHLN-GNN by +6%/+3.9%@F1 through optimized time information embedding; RF and CTDM, as the better traditional machine learning algorithms, still achieves +7.1%/+2.7%@F1 improvement over Balanced-BiEGCN, indicating that incorporating node relationships and dynamic information effectively enhances detection robustness. In dynamic graph scenarios, EvolveGCN-O and EvolveGCN-H belong to dynamic graph neural networks, while GRU and LSTM belong to recurrent neural networks. EvolveGCN-O performs better, highlighting the critical role of feature mining in detection; Balanced-BiEGCN achieves a +4.5%@F1 improvement over EvolveGCN-O. Overall, the advantages of Balanced-BiEGCN stem from two innovations: first, it alleviates class imbalance by converting normal nodes into new abnormal nodes; second, it enhances the ability to mine feature dependencies in long-term subgraphs, significantly improving performance in anomaly detection tasks and validating the effectiveness of integrating dynamic temporal information with graph structure modeling.

**Figure 4:**
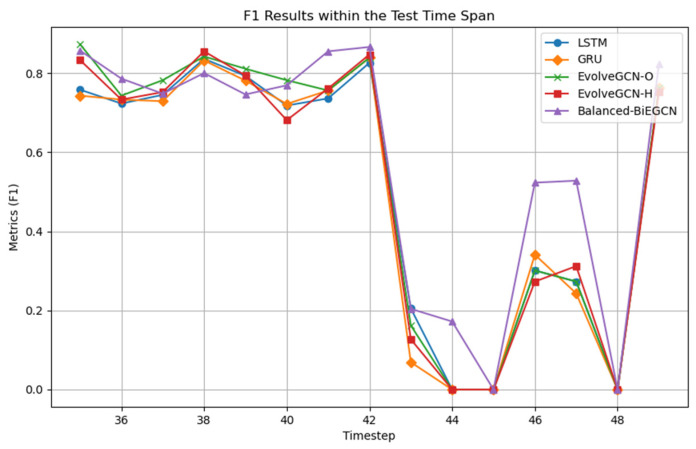
Dynamic graph approach for illegal F1 results in the 35–49 time range.

In addition, to explore the detection capabilities of the five dynamic graph approaches in Table 1 at different time points, this section selects subgraphs from time points 35 to 49 for comparison experiments. The results are shown in Figure 4. The experiment shows that in the time interval 35–42, all methods show good detection performance; however, starting from time point 43, the model performance fluctuates. It is worth noting that Balanced-BiEGCN has a significant advantage in difficult-to-detect subgraphs such as 44, 46, and 47, which fully demonstrates the advantages of this model in handling hard abnormal samples.

### 4.4. Ablation Study

To better demonstrate the effectiveness of Balanced-BiEGCN and the impact of each innovative component, this section will conduct ablation experiments using the test set.

The following components are removed: (1) Base (EvolveGCN-O); (2) Base + Bi-EvolveGCN (replacing the EvolveGCN in Base with Bi-EvolveGCN); (3) Balanced-BiEGCN—$L_{ada}$ (removing loss function $L_{ada}$ from Balanced-BiEGCN); (4) Balanced-BiEGCN—$L_{ssa}$ (removing Balanced-BiEGCN with loss function $L_{ssa}$); (5) Balanced-BiEGCN.

According to the experimental data in Table 2, the Balanced-BiEGCN significantly outperforms the baseline method in key metrics, achieving improvements of +4.5%@F1, +2.9%@Pre, and +5.1%@Re. Further analysis of the functionality of each module reveals that modules (2), (3), and (4) contribute +1.5%, +3.3%, and +2.4%@F1 improvements over the Base model, respectively. These figures clearly demonstrate the contribution of each component to the overall model performance. First, the node feature fusion strategy under the bidirectional temporal update parameter mechanism effectively improves the model’s ability to mine deep node features by strengthening feature interactions in the temporal dimension. This can effectively provide richer features for the subsequent generation of pseudo-anomalous nodes. Second, adjacency aggregation degree loss plays a key role in the node conversion process. Based on the explicit modeling of structural dependencies between nodes, it optimizes the conversion quality of normal nodes to new abnormal nodes. Finally, symmetric space adjustment loss enhances the model’s ability to distinguish between fuzzy class boundaries by spatially aligning the novel abnormal node class with real hard-to-mine abnormal node classes. Through mechanism innovation and complementary functions, the above modules jointly contribute to the performance advantages of Balanced-BiEGCN in complex anomaly detection scenarios.

### 4.5. Quantitative Experiment

**Figure 5:**
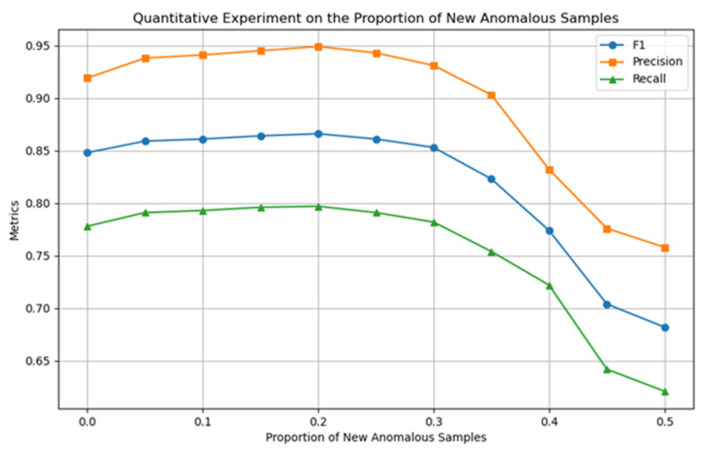
Quantitative experiment of pseudo-anomaly sample ratio ε.

**Figure 6:**
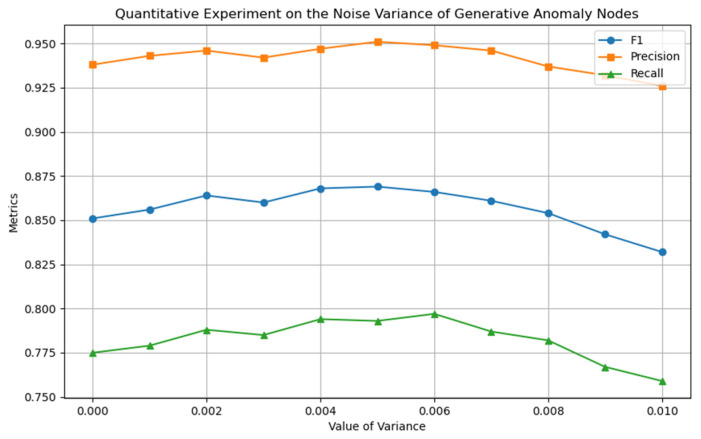
Quantitative experiment of noise variance parameter σ.

**Figure 7:**
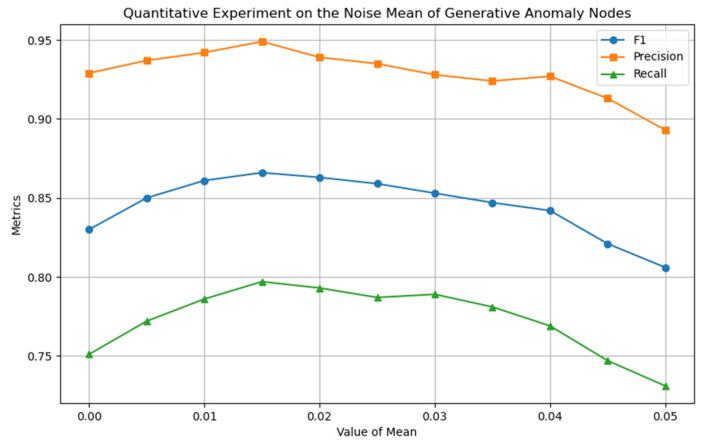
Quantitative experiment of noise mean parameter μ.

This section uses quantitative experimental strategies to optimize the model’s core hyperparameters and determine their optimal configuration. Figure 5, Figure 6 and Figure 7 show the experimental results for the four key hyperparameters, followed by a detailed analysis of the design logic and output characteristics of each experiment.

This section conducts a quantitative analysis of the impact of parameter ε (defined in Section 3.2) on the classification accuracy of the model for the proportion of newly generated abnormal samples. As shown in Figure 5, when ε=0.2, the classification accuracy of the model reaches its optimal level; after exceeding this threshold, as the proportion of pseudo-abnormal samples increases, the model performance shows a significant decline, so ε=0.2 is determined.

To construct a symmetrical feature distribution between novel abnormal node classes and normal node classes, this study introduced noise perturbations to normal nodes to generate a guiding feature set, thereby highlighting the core regulatory role of noise parameters in the construction of pseudo-abnormal samples. The experiment focused on the noise variance σ (Equation (5)), which, as the core factor regulating the feature distribution range of novel abnormal node classes, directly determines the distribution fit between novel abnormal and real abnormal node classes. As shown in Figure 6, when σ=0.005 is set, the model performance is optimal, indicating that this variance setting achieves a reasonable balance between novel abnormal nodes in the feature space of normal nodes and real abnormal nodes. We analyzed the impact of the mean parameter μ on model performance. This parameter determines the degree of deviation of the generated novel abnormal node class relative to the normal node class in the feature space. As shown in Figure 7, the model achieves the peak F1 value at μ=0.015, indicating that the distribution center of novel abnormal nodes is highly consistent with the feature space position of real abnormal nodes under this parameter setting, effectively enhancing the model’s learning ability for abnormal nodes. In summary, the model achieves the optimal state at σ=0.005 and μ=0.015. The feature space distribution of novel abnormal samples generated under this parameter combination has the smallest semantic distance from real abnormal samples, providing more effective learning information for the model.

**Figure 8:**
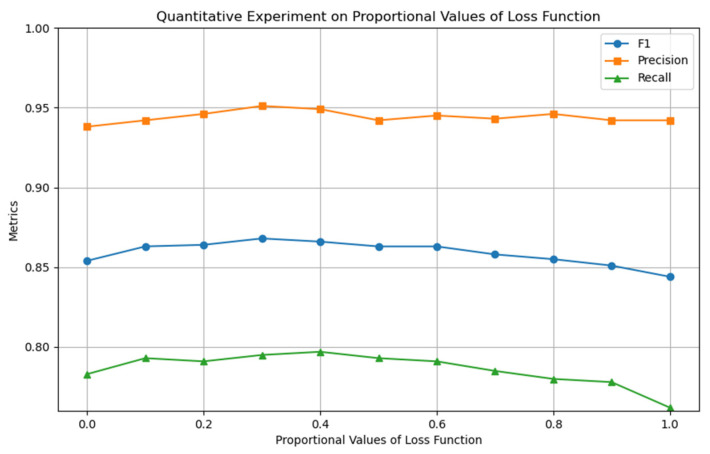
Quantitative experiment of loss function ratio l.

In the model training phase, optimization experiments were designed around the weight parameter l of the loss function (Equation (12)), to coordinate the optimization contributions of multiple loss terms. As shown in Figure 8, the model performance reached its optimal state when l=0.4 was set, verifying the coordination ability of this weight in optimizing multiple loss terms.

### 4.6. Visualization Experiment

**Figure 9:**
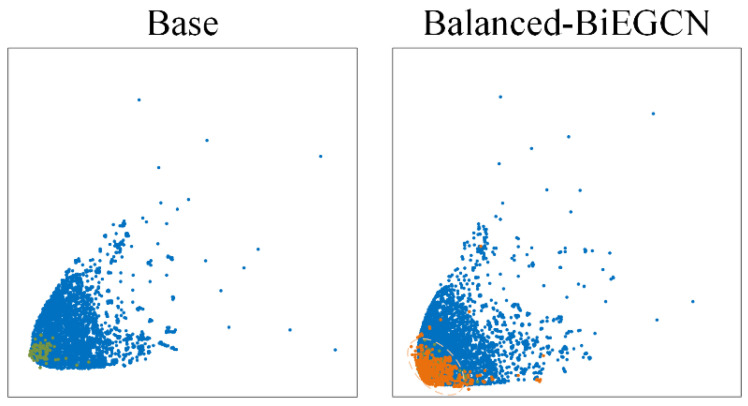
Visualization experiment of Balanced-BiEGCN and base methods.

To further investigate the performance characteristics of Balanced-BiEGCN in generating novel abnormal nodes, this experiment selected the subgraph at time step 1 from the Elliptic dataset for visualization and employed the t-SNE [42] dimension reduction algorithm to visualize the feature space. By comparing the performance of the baseline method and the Balanced-BiEGCN method in Figure 9, normal nodes are marked with blue circles, original abnormal nodes are labeled with green circles, and the novel generated abnormal nodes by Balanced-BiEGCN are represented by orange circles. The light orange dashed box outlines the spatial distribution area of the novel generated abnormal nodes.

From the comparison results in Figure 9, it can be seen that the spatial distribution of all nodes in the benchmark method forms a clear boundary range, and abnormal nodes are mostly concentrated in the spatial area with the highest node density. The cluster area of the novel generated abnormal nodes in Balanced-BiEGCN is close to that of the real abnormal nodes, mainly distributed in the lower left of the overall area. Although the distribution of the novel generated abnormal nodes is mostly within the distribution range of normal nodes, further observation shows that their boundaries are deeper in terms of spatial distribution.

In summary, the novel generated abnormal nodes produced by Balanced-BiEGCN are highly similar to the real abnormal node set in terms of spatial distribution characteristics and are more difficult to distinguish, demonstrating strong concealment and mining difficulty. This characteristic provides more challenging training samples for the model, effectively improving its ability to identify anomalies in complex scenarios.

## 5. Conclusions and Outlook

This paper tackles two critical challenges in Bitcoin transaction anomaly detection: the inadequate modeling of temporal dependencies in dynamic graphs and the persistent issue of class imbalance. To address these challenges, we propose the Balanced-BiEGCN, a novel framework that combines the bidirectional EvolveGCN model with advanced class-balanced learning techniques. Our method improves temporal dependency modeling by leveraging a Bi-EvolveGCN architecture that embeds bidirectional temporal subgraphs. This enhances the capture of long-term dependencies in the complex transaction network dynamics of Bitcoin.

In the node classification phase, we introduce the innovative Classifier based on Sample Class Transformation (CSCT). This, paired with our novel symmetric space adjustment loss function and adjacency distance adaptive loss function, facilitates the generation of abnormal samples by transforming normal nodes, thereby effectively balancing the class distribution. This dual approach not only enhances anomaly detection but also overcomes the limitations posed by the scarcity of labeled anomaly data.

Our findings make significant contributions to the field by offering a more robust and scalable method for handling the evolving nature of Bitcoin transactions. This work also presents a promising direction for future research, where models could be developed that require only labeled normal samples, further simplifying and broadening the applicability of anomaly detection. This shift toward minimal supervision holds the potential to revolutionize how anomaly detection is approached in dynamic and imbalanced datasets, making it more accessible, scalable, and applicable to various real-world applications beyond Bitcoin transactions.

Looking ahead, we foresee several potential avenues for further exploration. Future models could integrate more sophisticated temporal dependency structures and fine-tune the generation of abnormal samples to capture increasingly subtle anomalies. Additionally, the framework could be extended to other cryptocurrency networks and dynamic systems with similar challenges, creating a broader impact in anomaly detection for financial and security applications. Thus, the work presented not only addresses current limitations but also opens new pathways for scalable, low-labeled anomaly detection in complex and evolving networks.

## Figures and Tables

**Table 1:** Comparison experiments between Balanced-BiEGCN and mainstream methods.

Learning Type	Method	Illicit		
		F1	Pre	Re
Static graph	GCN [36]	0.628	0.812	0.512
	Skip-GCN [37]	0.705	0.812	0.623
	GraphSAGE [38]	0.634	0.537	0.465
	GraphSMOTE [16]	0.582	0.562	0.604
	MLP [33]	0.653	0.694	0.617
	RF [39]	0.694	0.803	0.611
	HHLN-GNN [40]	0.726	0.842	0.638
Dynamic graph	GRU [31]	0.682	0.661	0.728
	LSTM [25]	0.691	0.643	0.734
	EvolveGCN-H [14]	0.714	0.824	0.631
	EvolveGCN-O [14]	0.720	0.850	0.624
	CTDM [41]	0.738	0.851	0.652
	Balanced-BiEGCN	0.765	0.879	0.675

**Table 2:** Ablation experiments of Balanced-BiEGCN.

	Datasets	Illicit		
Method		F1	Pre	Re
Base		0.72	0.85	0.624
Base+Bi-EvolveGCN		0.737	0.861	0.644
Balanced-BiEGCN—$L_{ada}$		0.753	0.868	0.665
Balanced-BiEGCN—$L_{ssa}$		0.744	0.872	0.649
Balanced-BiEGCN		0.765	0.879	0.675

## Data Availability

The datasets used in this study are publicly available: Elliptic, www.elliptic.co.
